# Multisystem Analysis of *Mycobacterium tuberculosis* Reveals Kinase-Dependent Remodeling of the Pathogen-Environment Interface

**DOI:** 10.1128/mBio.02333-17

**Published:** 2018-03-06

**Authors:** Xavier Carette, John Platig, David C. Young, Michaela Helmel, Albert T. Young, Zhe Wang, Lakshmi-Prasad Potluri, Cameron Stuver Moody, Jumei Zeng, Sladjana Prisic, Joseph N. Paulson, Jan Muntel, Ashoka V. R. Madduri, Jorge Velarde, Jacob A. Mayfield, Christopher Locher, Tiansheng Wang, John Quackenbush, Kyu Y. Rhee, D. Branch Moody, Hanno Steen, Robert N. Husson

**Affiliations:** a Division of Infectious Diseases, Boston Children’s Hospital/Harvard Medical School, Boston, Massachusetts, USA; b Department of Biostatistics and Computational Biology, Dana-Farber Cancer Institute, Boston, Massachusetts, USA; c Department of Biostatistics, Harvard T.H. Chan School of Public Health, Boston, Massachusetts, USA; d Division of Rheumatology, Brigham and Women’s Hospital/Harvard Medical School, Boston, Massachusetts, USA; e Department of Pathology, Boston Children’s Hospital/Harvard Medical School, Boston, Massachusetts, USA; f Department of Medicine, Weill Cornell Medical College, New York, New York, USA; g Vertex Pharmaceuticals Incorporated, Boston, Massachusetts, USA; Washington University in St. Louis School of Medicine

**Keywords:** *Mycobacterium tuberculosis*, PknB, Ser/Thr protein kinase, signal transduction, two-component regulatory systems

## Abstract

Tuberculosis is the leading killer among infectious diseases worldwide. Increasing multidrug resistance has prompted new approaches for tuberculosis drug development, including targeted inhibition of virulence determinants and of signaling cascades that control many downstream pathways. We used a multisystem approach to determine the effects of a potent small-molecule inhibitor of the essential *Mycobacterium tuberculosis* Ser/Thr protein kinases PknA and PknB. We observed differential levels of phosphorylation of many proteins and extensive changes in levels of gene expression, protein abundance, cell wall lipids, and intracellular metabolites. The patterns of these changes indicate regulation by PknA and PknB of several pathways required for cell growth, including ATP synthesis, DNA synthesis, and translation. These data also highlight effects on pathways for remodeling of the mycobacterial cell envelope via control of peptidoglycan turnover, lipid content, a SigE-mediated envelope stress response, transmembrane transport systems, and protein secretion systems. Integrated analysis of phosphoproteins, transcripts, proteins, and lipids identified an unexpected pathway whereby threonine phosphorylation of the essential response regulator MtrA decreases its DNA binding activity. Inhibition of this phosphorylation is linked to decreased expression of genes for peptidoglycan turnover, and of genes for mycolyl transferases, with concomitant changes in mycolates and glycolipids in the cell envelope. These findings reveal novel roles for PknA and PknB in regulating multiple essential cell functions and confirm that these kinases are potentially valuable targets for new antituberculosis drugs. In addition, the data from these linked multisystems provide a valuable resource for future targeted investigations into the pathways regulated by these kinases in the *M. tuberculosis* cell.

## IMPORTANCE

Tuberculosis is the leading killer among infectious diseases worldwide. Increasing drug resistance threatens efforts to control this epidemic; thus, new antitubercular drugs are urgently needed. We performed an integrated, multisystem analysis of *Mycobacterium tuberculosis* responses to inhibition of its two essential serine/threonine protein kinases. These kinases allow the bacterium to adapt to its environment by phosphorylating cellular proteins in response to extracellular signals. We identified differentially phosphorylated proteins, downstream changes in levels of specific mRNA and protein abundance, and alterations in the metabolite and lipid content of the cell. These results include changes previously linked to growth arrest and also reveal new roles for these kinases in regulating essential processes, including growth, stress responses, transport of proteins and other molecules, and the structure of the mycobacterial cell envelope. Our multisystem data identify PknA and PknB as promising targets for drug development and provide a valuable resource for future investigation of their functions.

## INTRODUCTION

Phosphorylation-based signal transduction allows adaptation to environmental conditions by linking extracellular signals to intracellular regulatory mechanisms. In most bacteria, the dominant mechanism of phosphorylation-based transmembrane signaling is the two-component system (1), where ligand binding by the transmembrane sensor protein initiates a phosphorelay to the cognate intracellular response regulator protein, which is typically a transcription factor that controls expression of specific genes. Bacteria also use receptor-type Ser/Thr protein kinases (STPKs), which have an extracytoplasmic receptor domain and an intracellular kinase domain that is structurally similar to eukaryotic Ser/Thr kinase domains (2, 3). In response to an extracellular signal, the cytoplasmic kinase domain phosphorylates substrate proteins, including transcription factors, structural proteins, and enzymes. Phosphorylation of these proteins then triggers a cascade of downstream changes in levels of gene expression, proteins, small molecules, and lipids, broadly altering physiological systems in the cell.

The success of kinase inhibitors as drugs to treat human diseases (4) has stimulated interest in developing kinase inhibitors as a new class of antibacterial drugs. With tuberculosis killing more people worldwide than any other infectious disease, together with increasing rates of drug resistance, there is a strong rationale to develop drugs that inhibit new bacterial targets. PknA and PknB are potentially valuable targets for antituberculosis drug development based on their essentiality for growth *in vitro* and in mice (5–7) and on the successful development of several kinase inhibitors as drugs to treat human disease (4).

Targeted research has provided insights into a number of likely functions of PknA and PknB. Genes for both kinases are present in an operon that includes genes for a Ser/Thr phosphatase (*pstP*), a candidate peptidoglycan (PG) synthase (*rodA*) (8), and a class B penicillin-binding protein (*pbpA*). Phosphorylation mediated by PknA and PknB controls activity of PG synthesis regulators (9–11), and peptidoglycan fragments have been shown to bind to conserved motifs in the extracytoplasmic domain of PknB and its orthologue in *Bacillus subtilis* to regulate cell growth and morphology (12, 13). PknB also regulates central carbon metabolism in *Mycobacterium tuberculosis* through phosphorylation of the GarA regulatory protein (14, 15). Ser/Thr phosphorylation also regulates the activity of MabA, KasB, and InhA, essential enzymes involved in mycolic acid synthesis in *M. tuberculosis* (16–18). Despite these insights, we lack an integrated understanding of the effects of PknA and PknB on *M. tuberculosis* cell physiology.

To develop a more comprehensive view of the functions of PknA and PknB, we used a newly developed small-molecule inhibitor of these kinases together with unbiased multisystem profiling. Compared to genetic strategies that rely on protein depletion, the advantage of chemical inhibition is the rapid inhibition of enzyme activity, which allows identification of both early, direct effects on protein phosphorylation and downstream, indirect effects of blocking kinase activity. Given the potential for PknA and PknB to broadly regulate multiple cellular pathways, we performed phosphoproteomics, proteomics, transcriptomics, lipidomics, and metabolomics analyses. This approach identified changes in protein phosphorylation in response to kinase inhibition that were linked to broad downstream changes in gene expression, protein abundance, and lipid and metabolite profiles. Integration of these data identifies changes in pathways for cell growth, remodeling of the mycobacterial cell envelope, protein and small-molecule transport, and stress responses and reveals an unexpected intersection of PknA/PknB-mediated signal transduction with an essential two-component system.

## RESULTS

### Potent chemical inhibition of PknA and PknB kinase activity inhibits *M. tuberculosis* growth.

**FIG 1:**
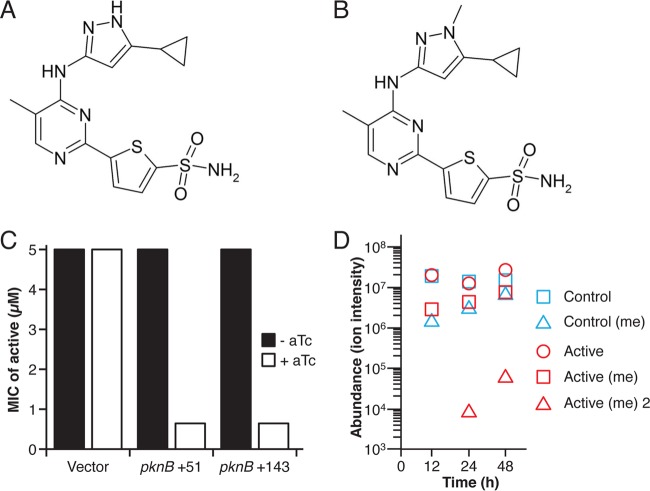
Kinase inhibitor structure and activity and entry into *M. tuberculosis* cells. (A and B) Structures of the active kinase inhibitor (A) and the inactive analogue (B) used in this work. (C) MIC shift in response to *pknB* depletion by CRISPR interference targeting 2 sites in *pknB* (+51 and +143 relative to the initiation codon). MICs were measured with the microplate alamarBlue assay. The MIC of the kinase inhibitor decreased from 5 μM in the control to 0.63 μM in both kinase-depleted strains under conditions (with aTc [anhydrotetracycline] added to induce CRISPR interference) that achieved ~90% decreased *pknB* expression (*n* = 2 for each targeting construct; *n* = 4 total). (D) Time-dependent accumulation and metabolism of the active and control compounds following incubation of *M. tuberculosis* cells on Middlebrook 7H9 agar containing 10 μM compound. *y*-axis values denote abundances of compounds recovered from *M. tuberculosis* cells as indicated by ion intensity. Red symbols indicate active inhibitor (*m*/*z* = 376.078) and transformation products corresponding to monomethylated (*m*/*z* = 390.093) [Active (me)] and dimethylated (*m*/*z* = 404.109) [Active (me) 2] adducts. Blue symbols indicate the control compound (*m*/*z* = 390.093) and the transformation product corresponding to monomethylated adduct (*m*/*z* = 404.109) [Control (me)]. All values represent means ± standard errors of the means (SEM) of results from experimental triplicates (*n* = 3) and are representative of results from 3 independent experiments.

From a series of small molecules developed by Vertex Pharmaceuticals Incorporated to inhibit PknA and PknB, we selected a 5-substituted pyrimidine (Fig. 1A) based on its potent *in vitro* inhibition of PknB (inhibitor constant *K*_*i*_ = 0.004 μM) and PknA (*K*_*i*_ = 0.018 μM), its ability to inhibit growth of *M. tuberculosis* H37Ra [minimal inhibitory concentration (MIC) = 4.5 μM], and its selectivity for PknA and PknB relative to human kinases (19). An analogue that differs only by one additional N-methyl group (Fig. 1B) showed no inhibition of PknA or PknB kinase activity (*K*_*i*_ = >5 μM) or *M. tuberculosis* growth (MIC, >100 μM) and served as a control in all experiments.

We used this inhibitor and the inactive analogue to investigate PknA and PknB function in detail in the virulent H37Rv strain. We determined the MIC of the active compound to be 5 μM and that of the inactive analogue to be >160 μM. To test whether inhibition of PknB activity is responsible for *M. tuberculosis* growth inhibition, we measured the MIC of the active compound in a strain in which *pknB* expression was reduced by 80% to 90%, using clustered regularly interspaced short palindromic repeat (CRISPR) interference (20). We observed an 8-fold increase in susceptibility to the inhibitor in the PknB-depleted strain, with a shift of the MIC from 5 μM to 0.63 μM (Fig. 1C). Consistent with PknA being in the same pathway as PknB, knockdown of both *pknA* expression and *pknB* expression showed similar (4- to 8-fold) MIC shifts (data not shown). These data indicate that a major part of the growth inhibitory activity of the active compound results from inhibiting PknB and PknA, the only two essential *M. tuberculosis* STPKs, and strongly support the hypothesis that physiological changes in responses to the inhibitor result from inhibition of these kinases.

Because the kinase domains of some other *M. tuberculosis* STPKs are similar to those of PknB and PknA (21), we tested the inhibitor against seven other *M. tuberculosis* STPKs for which recombinant protein could be produced (see Table S1 in the supplemental material). In addition to PknA and PknB, this compound potently inhibits the nonessential kinases PknD and PknL *in vitro*. Two candidate PknD substrates, Rv0516 and Rv3701, which are also phosphorylated by PknB and by PknA and PknB, respectively (22, 23), and one candidate PknL substrate, Rv2175c (only shown to be phosphorylated *in vitro*) (24), have been previously identified. Though these proteins were present in our proteomics data, we did not detect phosphorylation of these proteins in our data. Analysis of a *pknD* overexpression strain identified 7 highly differentially expressed genes (22). Expression of five of these genes in our transcriptomic data was not consistent with PknD inhibition. In a *pknL* knockdown strain, 7 proteins were highly expressed compared to the wild-type results (25); in our kinase inhibition data, none of these showed significantly increased expression.

10.1128/mBio.02333-17.4 TABLE S1  Activity of kinase inhibitor and inactive control compounds against *M. tuberculosis* STPKs. Download TABLE S1, DOCX file, 0.04 MB. Copyright © 2018 Carette et al. 2018 Carette et al. This content is distributed under the terms of the Creative Commons Attribution 4.0 International license.

We also observed significantly decreased phosphorylation of phosphopeptides from PknA and PknB, and from known PknA/PknB substrates in extracts of cells treated with the inhibitor, that indicated that PknA and PknB are primary targets of the inhibitor (see "Data analysis—phosphoproteomics and proteomics" section below). In contrast, phosphorylation of peptides from PknD, which also autophosphorylates, was stable or increased and PknL phosphopeptides were not detected in any sample. Phosphorylation of phosphopeptides from PknE, PknF, and PknG was also stable or increased, and phosphopeptides from PknH, PknI, PknJ, and PknK were not detected. These data support the conclusion that major effects of the inhibitor result from inhibition of PknA and PknB and do not support the identification of PknD or PknL as targets of the kinase inhibitor.

We further investigated whether inhibition of PknD or PknL might account for some effects of the kinase inhibitor by performing transcriptomics analysis of inhibitor-treated *pknD* and *pknL* deletion strains. Global gene expression in the inhibitor-treated mutants was highly correlated with the wild-type results (Pearson’s *r* = 0.71 for *pknD* and 0.78 for *pknL*). To the extent that kinase gene deletion and kinase inhibition have similar effects, for a gene whose expression is affected by PknD or PknL kinase activity, if the inhibitor blocks this activity, we would expect (i) that inhibitor effects on gene expression in the wild type would not be seen in the mutant treated with the inhibitor and (ii) that expression of the gene in the mutant treated with the control compound would be similar to its expression in the wild type treated with the inhibitor.

Of the 298 highly differentially expressed genes in the wild type treated with the inhibitor (log_2_ fold change [Log_2_FC], >1 or ≤1; *P* < 0.0001) (Tables S2B and C), 255 (86%) and 250 (84%) were similarly differentially expressed (Log_2_FC, >0.5 and ≤0.5; *P* < 0.01) in the inhibitor-treated *pknD* and *pknL* mutants, respectively (Table S2D). Lower cutoff values were used because fewer replicates (*n* = 3) were performed in the experiments comparing mutant strains to the wild type. After excluding phage genes, expression of 27 (9.1%) and 35 (11.7%) genes (i) did not change in the inhibitor-treated *pknD* or *pknL* strains, respectively, in a manner similar to that seen with the inhibitor-treated wild-type strain and (ii) changed significantly (Log_2_FC, >0.5 or ≤0.5; *P* < 0.01) in the control-treated *pknD* and *pknL* strains in a manner similar to that seen with the inhibitor-treated wild type. These criteria identified genes whose expression might be affected by inhibition of PknD or PknL activity by the kinase inhibitor. Given that distinct signal transduction systems often have overlapping downstream pathways, however, this likely represents a substantial overestimate of such effects. Taken together, these several analyses strongly support the identification of PknA and PknB as primary targets of the kinase inhibitor and the supposition that inhibition of these two kinases is the main source of the changes obtained in our multisystem data.

10.1128/mBio.02333-17.5 TABLE S2  Expression ratio for all genes of RNA from samples treated with the kinase inhibitor to samples treated with the inactive control compound (A), genes with significantly increased expression and comparative gene expression from other conditions (B), genes with significantly decreased expression and comparative gene expression from other conditions (C), and comparison of highly regulated genes to their expression in *pknD* and *pknL* deletion strains (D). Download TABLE S2, XLSX file, 0.6 MB. Copyright © 2018 Carette et al. 2018 Carette et al. This content is distributed under the terms of the Creative Commons Attribution 4.0 International license.

### Entry, activity, and metabolism of the kinase inhibitor in *M. tuberculosis* cells.

We sought to understand kinase effects on cellular functions in a manner that would be minimally confounded by cell death pathways. Pilot studies showed growth inhibition but minimal cell death at an inhibitor concentration of 4-fold the MIC (20 μM) (see Fig. S1 in the supplemental material). Serial samples (0, 12, 24, and 48 h) allowed kinetic analysis to identify early and late effects. Experiments were repeated two to three times with three biological replicates in each experiment. Samples were processed in parallel for quantitative transcriptomic, proteomic, phosphoproteomic, lipidomic, and metabolomic analyses by the participating laboratories. Results are reported as log_2_-fold change (Log_2_FC) in inhibitor-treated versus control-treated cells, with *P* values adjusted for multiple testing.

10.1128/mBio.02333-17.1 FIG S1  Survival of *M. tuberculosis* after incubation with inhibitor and control compounds. *M. tuberculosis*-laden filters were prepared as described in Materials and Methods. The filters were directly placed on 7H9 agar plus ADNC plus inhibitor and incubated at 37°C. After 2 days, the bacteria were harvested and suspended in phosphate-buffered saline (PBS)–0.05% Tween 80. Serial 10-fold dilutions (10^−1^ to 10^−5^) were prepared, and 10 µl of each dilution was spotted on 7H9 agar plus ADNC and incubated for 3 weeks at 37°C. Download FIG S1, DOCX file, 0.4 MB. Copyright © 2018 Carette et al. 2018 Carette et al. This content is distributed under the terms of the Creative Commons Attribution 4.0 International license.

Unbiased mass spectrometry (MS)-based metabolomics analysis of cell extracts detected inhibitor and control compounds in cell extracts at the earliest time point (12 h) (Fig. 1D). Ions indicating that the compounds underwent one or two N-methylation events within the bacteria were also identified at 12 to 24 h, and the levels increased over time. The intensity of ions corresponding to the unmodified inhibitor remained stable, however, indicating that compound delivery from the medium was sufficient to maintain growth inhibitory concentrations at all time points.

### Kinase inhibition identifies the mycobacterial cell envelope as a major target of PknA and PknB.

**FIG 2:**
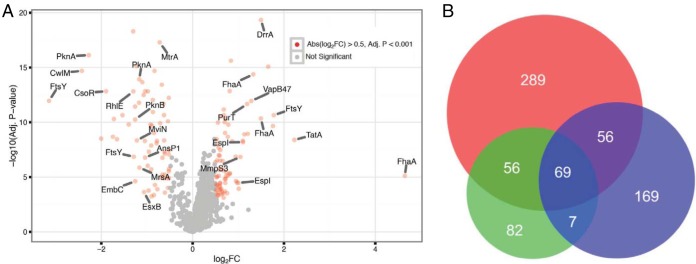
Differential protein phosphorylation in response to PknA/PknB inhibition. (A) Volcano plot of identified phosphopeptides shown as the ratio of their abundance in inhibitor-treated samples to that in control samples. Significantly differentially abundant phosphopeptides (Log_2_FC, >0.5 or ≤0.5; adjusted [Adj.] *P* < 0.001) are shown in red. Data are from 2 independent experiments, both performed with biological triplicates (*n* = 6) (B) Venn diagram showing overlap of the phosphoproteins identified in this work (shown in red) (*n* = 470) and our prior results (21) (shown in blue) (*n* = 301) and results from another recent *M. tuberculosis* phosphoproteomic study (26) (shown in green) (*n* = 214).

In two experiments performed with three biological replicates each, we identified a total of 1,373 phosphopeptides corresponding to 1,241 unique phosphorylation sites on 470 proteins (Fig. 2A; see also Table S3A in the supplemental material). Although these results partially overlap those from prior studies (21, 26–28) (Fig. 2B), many new phosphoproteins were identified, substantially expanding the *M. tuberculosis* phosphoproteome. Analysis of these phosphorylation sites identified eight significant motifs (*P* < 0.01), comprising four each with Ser or Thr as the phosphorylated residue (Fig. S2A). Three of these, Thr(P)-X-Pro, Pro-Thr(P), and Glu-X-X-T(P), were also present in our prior *M. tuberculosis* phosphoproteomic data (21).

10.1128/mBio.02333-17.2 FIG S2  (A) Significant phosphorylation site motifs identified among all unique phosphopeptides. (B) Significant motifs from phosphopeptides that showed significantly decreased phosphorylation in response to the kinase inhibitor. Download FIG S2, EPS file, 0.8 MB. Copyright © 2018 Carette et al. 2018 Carette et al. This content is distributed under the terms of the Creative Commons Attribution 4.0 International license.

10.1128/mBio.02333-17.6 TABLE S3  List of all detected phosphopeptides (A), distribution of phosphorylated residues (B), quantified phosphopeptides (C), phosphopeptides with significantly decreased expression (D), phosphopeptides with significantly increased expression (E), and time course of quantified phosphopeptides (F). Download TABLE S3, XLSX file, 0.4 MB. Copyright © 2018 Carette et al. 2018 Carette et al. This content is distributed under the terms of the Creative Commons Attribution 4.0 International license.

Applying change criteria of Log_2_FC values of ≤0.5 and *P* values of <0.001 to phosphopeptides detected in >7 of 36 samples, 68 phosphopeptides from 48 proteins showed decreased phosphorylation in response to treatment with the inhibitor (Table S3C and D). Decreases in the level of phosphorylation were maximal or nearly maximal at the earliest time point (12 h) for many of these proteins, which is consistent with their being direct *in vivo* substrates of PknA and/or PknB (Table S3F). Notably, Thr was the phosphorylated residue in 88% of the phosphopeptides that were significantly decreased in level versus 48% overall, consistent with the preference of PknB and PknA for Thr phosphorylation (21, 27). The Glu-X-X-Thr(P) and Thr(P)-X-Pro motifs were significantly associated with decreased Thr phosphorylation (Fig. S2B). We identified phosphoproteins in all nine functional groups of the H37Rv genome annotation (29). Proteins with decreased phosphorylation were significantly enriched in “cell walls and cell processes” (Fisher’s exact test, *P* < 0.0001) and significantly depleted in “intermediary metabolism and respiration” (*P* = 0.001) (29). Thirty (63%) of the proteins with decreased phosphorylation were membrane proteins, secretion system proteins, or regulators of secretion or cell wall synthesis.

Significantly decreased phosphorylation (Table S3D) was seen in peptides from PknA and PknB and from regulators of PG synthesis previously shown to be substrates of PknA and/or PknB, including Rv3915 (CwlM, which activates the first committed step of PG synthesis when phosphorylated [11]), Rv0020 (FhaA [forkhead domain-associated protein A]), and Rv3910 (MviN, which interacts with FhaA to regulate PG synthesis when phosphorylated [9]). Decreased phosphorylation was also seen in proteins involved in cell division, including Rv2748c (FtsK, an essential membrane protein involved in chromosome segregation during cell division) and Rv2151c (a homologue of FtsQ, an essential membrane protein involved in septum formation). Proteins from three protein secretion pathways also showed significantly decreased phosphorylation. These included general secretion system components Rv0638 (SecE, an essential preprotein translocase), Rv2586c (SecF, an essential component of the Sec translocase machinery), and Rv2921c (FtsY, the signal recognition particle receptor); the twin arginine transport system component Rv2094 (TatA, the essential channel-forming protein); and ESX-1 type VII secretion system protein Rv3874 (EsxB, an antigen and virulence factor). These data thus suggest that PknA and PknB directly regulate the mycobacterial cell wall, cell division, and protein secretion systems that export distinct classes of proteins to the periplasm and surface of the mycobacterial cell envelope, the interface of this pathogen with the host.

Seventy-eight phosphopeptides derived from 48 proteins showed significantly increased phosphorylation (Table S3C and E), likely representing activity of kinases downstream of PknA and PknB. Proteins with increased phosphorylation included several chaperone/heat shock proteins and, in a few cases, the same proteins as those that had decreased phosphorylation, including FhaA, FtsY, and PknA. Proteins with increased phosphorylation were significantly enriched in “virulence, detoxification, and adaptation” phosphoproteins (*P* = 0.01) and depleted in “information pathway” phosphoproteins (*P* = 0.01). In contrast to the low proportion (12%) of Ser residues that showed decreased phosphorylation, a much higher proportion (44%) of phosphopeptides with increased phosphorylation were phosphorylated on Ser (Table S3D and E).

We also identified Tyr phosphorylation of 39 peptides, which has been shown to be mediated by PknB (30), from 24 proteins (Table S3A and B), of which 17 (44%) were chaperone protein such as GroEL2 and DnaK. Two sites of Tyr phosphorylation in the PG synthesis regulator FhaA showed significantly decreased phosphorylation in kinase inhibitor-treated samples (Table S3D), further supporting regulation of PG synthesis by PknB.

### PknA and PknB inhibition leads to transcriptome remodeling affecting pathways for growth, cell envelope structure, transport, and stress response.

**FIG 3:**
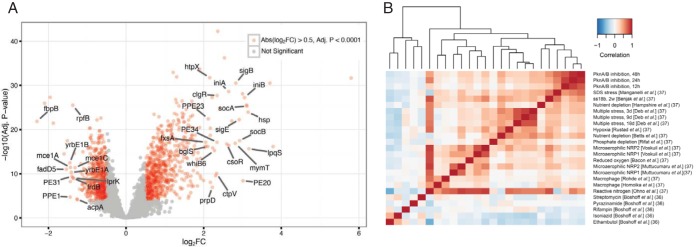
Differential gene expression in response to PknA/PknB inhibition. (A) Volcano plot of *M. tuberculosis* mRNAs shown as the ratio of their abundance in kinase inhibitor-treated samples to that in control samples. Significantly differentially abundant gene transcripts (Log_2_FC, >0.5 or ≤0.5; *P* = <0.0001) are shown in red. Examples of the most differentially abundant annotated genes (Log_2_FC, >2.0 or ≤1.25) are labeled. Data are from 3 independent experiments, each performed with biological triplicates (*n* = 9). (B) Hierarchical clustering of correlations between data from this work and published transcriptomics data obtained following exposure of *M. tuberculosis* to different stresses or antibiotics. The differential levels of gene expression determined in each experiment were used to calculate correlations. Numbers in parentheses represent the references from which transcriptional profiling data were obtained. The cited work by Benjak et al. includes data from prior studies cited within that reference as indicated in panel B.

The transcriptome sequencing (RNA-Seq) results provided the most comprehensive view of changes in *M. tuberculosis* physiology in response to the kinase inhibitor. Focusing on the most highly regulated genes (Log_2_FC values of >1.0 or ≤1.0 and *P* values of <0.0001), expression of 253 genes increased and expression of 45 genes decreased (Fig. 3A; see also Table S2B and C). In contrast to the early decreases in protein phosphorylation, for most genes, changes in expression present at 12 h continued in the same direction and increased in magnitude over the 48-h time course. This time-dependent reprogramming of the transcriptome likely results from a cascade of downstream effects triggered by the inhibition of PknA/PknB-mediated phosphorylation events.

Pathway enrichment analysis of Gene Ontology (GO), KEGG, and manually curated pathways identified cellular processes that were significantly affected (Table S4). We observed decreased expression in pathways for (i) translation, including most genes encoding ribosomal proteins; (ii) PG catabolism, including endopeptidases encoded by Rv0950c and Rv2190c and lytic transglycosylases encoded by resuscitation promoting factor (rpf) genes *rpfA*, *rpfB*, *rpfC*, and *rpfE* (*rpfD* was upregulated); (iii) the Mce-1 virulence-associated transport system, which plays a role in fatty acid import and affects the lipid content of the cell envelope (31, 32), and the Mce-4 transport complex, which imports cholesterol (33); (iv) oxidative phosphorylation pathways, including the proton-coupled type I NADH dehydrogenase (complex I), fumarate reductase and succinate dehydrogenase (complex II), and ATP synthase (complex V) pathways; (v) pathways for lipid synthesis, including fatty acid synthase II (FASII) enzymes required for mycolic acid synthesis; and (vi) mycolyl transferase genes *fbpA* and *fbpB*.

10.1128/mBio.02333-17.7 TABLE S4  RNA (A) and protein pathway (B) enrichment analysis. Download TABLE S4, XLSX file, 0.1 MB. Copyright © 2018 Carette et al. 2018 Carette et al. This content is distributed under the terms of the Creative Commons Attribution 4.0 International license.

Pathways with significantly increased expression included those involving (i) the SigE regulon, which is activated in response to cell envelope stress; (ii) mycobactin biosynthesis, which produces a secreted siderophore for iron acquisition; (iii) genes controlled by two copper-regulated repressors, CsoR and RicR, which derepress their regulons in response to increased copper concentrations (34, 35); (iv) the ESX1 type VII secretion system, which is required for virulence; (v) the twin arginine transport (TAT) protein secretion system; and (vi) eight MazF or VapC toxin genes. These examples, together with the many other affected pathways (Table S4), suggest a major role for PknA and PknB in broadly regulating *M. tuberculosis* physiology, in particular, the cell envelope, and the secretion and transport systems, which together define the interaction of the cell with the extracellular environment.

To examine the extent to which these effects are specific to kinase inhibition, we first compared our data to published transcriptome-wide analyses of gene expression in response to first-line tuberculosis drugs and to multiple stresses (36, 37). The transcriptome-wide response to kinase inhibition did not extensively overlap the prior results, consistent with the distinct mechanism of action of the kinase inhibitor (Fig. 3B). We then compared highly affected genes and pathways in our data to five data sets in the following categories of high relevance for tuberculosis treatment and pathogenesis: treatment with isoniazid, treatment with rifampin, macrophage infection, nutrient depletion, and hypoxia for 7 days (36, 38–40) (Tables S2B and S2C).

The hypoxia and nutrient depletion data and the kinase inhibitor data showed the greatest overlap. Pathways that showed substantial overlap for those two stress conditions included those associated with translation and ATP synthesis (both also decreased in rifampin treatment), type I NADH dehydrogenase, and succinate dehydrogenase (hypoxia only). Mce1 locus expression was highly decreased by hypoxia and partially decreased by nutrient depletion, macrophage infection, and rifampin exposure, whereas Mce4 gene expression was decreased in hypoxia and in macrophage infection. FASII gene expression was decreased during macrophage infection, *rpf* expression was decreased in hypoxia, and *rpfC* gene expression was decreased with rifampin treatment and in macrophages.

Among the pathways with increased transcription in response to kinase inhibition, the SigE regulon was upregulated under conditions of hypoxia and to a lesser extent under conditions of nutrient depletion and macrophage infection. Expression of ESX1 secretion system genes and the mycobactin biosynthetic pathway was not consistently increased under any condition. Expression of the CsoR and RicR copper-regulated operons was increased in response to hypoxia. Expression of four MazF/VapC toxin genes was increased under one other condition, and increased expression was specific to kinase inhibition for four genes. In summary, in our results, the data corresponding to transcriptomic changes that are required for growth and the hypoxia and starvation data showed substantial overlap, but many other changes were more specific to kinase inhibition (Tables S2B and S2C).

### Proteome changes partially parallel changes in gene expression.

Proteomics experiments were carried out in the data-independent acquisition mode (41) to maximize identification of proteins across multiple samples and thus increase statistical power for protein quantification. We detected 2,520 proteins across 72 samples. Applying change criteria of Log_2_FC values of >0.5 or ≤0.5 and *P* values of <0.001, expression levels of 110 proteins were significantly decreased and those of 82 proteins were significantly increased at 48 h of exposure to the kinase inhibitor (Table S5).

10.1128/mBio.02333-17.8 TABLE S5  Ratios of abundances of proteins from samples treated with the kinase inhibitor relative to samples treated with the inactive control compound (A), proteins with significantly increased abundance (B), and proteins with significantly decreased abundance (C). Download TABLE S5, XLSX file, 0.3 MB. Copyright © 2018 Carette et al. 2018 Carette et al. This content is distributed under the terms of the Creative Commons Attribution 4.0 International license.

Plotting the Log_2_FC data for each detected protein and the corresponding Log_2_FC data for each transcript (Fig. S3A), we observed a weak correlation between changes in levels of RNA and protein in response to the kinase inhibitor, a common finding that likely reflects posttranslational mechanisms that affect protein abundance. Plotting the protein Log_2_FC values corresponding to each RNA that was significantly changed in level (Fig. S3B) showed a similar pattern. Plotting the RNA Log_2_FC values for each protein that was significantly changed in level (Fig. S3C), however, showed a positive relation between the changes seen with most proteins and the corresponding transcript level changes. Several proteins that were markedly decreased in expression, however, had small changes in expression of the corresponding genes. The 5 proteins with the greatest decrease in expression (Rv3036c [antigen TB22.2], Rv3668c [a predicted protease], Rv1815 [a protein of unknown function], Rv3572 [a protein of unknown function], and Rv1887 [a possible protease]) were all experimentally verified to be secreted proteins, and all but Rv1887 have an N-terminal signal sequence (42) (Table S5). Additional secreted proteins, including Rv1926c (antigen Mpt63), Rv1980c (antigen mpt64), Rv1984 (a cutinase precursor), and Rv2376c (antigen CFP2), all of which have a signal sequence, were also markedly decreased in expression. The decrease in abundance of these proteins in the cell suggests altered activity of the general secretory pathway, potentially linked to the decreased phosphorylation of essential components of this pathway. Additional proteins with large decreases in expression in response to kinase inhibition included the secreted mycolyl transferases FbpB and FpbA and several membrane proteins and hypothetical proteins. These proteomic changes, together with the phosphoproteomic and transcriptomic data, provide multilevel support for the hypothesis that PknA and PknB regulate the *M. tuberculosis* cell envelope and protein secretion.

10.1128/mBio.02333-17.3 FIG S3  Plots showing Log_2_FC of RNA versus Log_2_FC of protein at 48 h of exposure to the kinase inhibitor. (A) Log_2_FC for all quantified proteins and the Log_2_FC for each corresponding mRNA. (B) Log_2_FC of all detected proteins for which the RNA met change criteria (Log_2_FC, >0.5 or ≤0.5; *P* < 0.0001). (C) Log_2_FC of RNAs for which the corresponding protein met change criteria (Log_2_FC, >0.5 or ≤0.5; *P* < 0.001). In panels B and C, the red dots indicate that both the RNA and protein data met the change criteria. Download FIG S3, TIF file, 0.1 MB. Copyright © 2018 Carette et al. 2018 Carette et al. This content is distributed under the terms of the Creative Commons Attribution 4.0 International license.

Pathway analysis (Table S4) identified significant increases in expression at 48 h for the SigE regulon and the ESX-1 secretion system, validating results of the RNA pathway enrichment analysis. We also noted enrichment of membrane proteins, PG assembly, and two-component signal transduction pathways, which were not enriched in the RNA pathway analysis. Pathways that showed significantly decreased protein abundance included NADH dehydrogenase activity and FASII pathways, both of which were similarly decreased in abundance in the RNA pathway analysis. Pathways for lipid biosynthesis and several metabolic pathways were decreased at the protein level but not in the RNA pathway analysis.

### Lipidomics identifies downstream effects of PknA and PknB inhibition.

**FIG 4:**
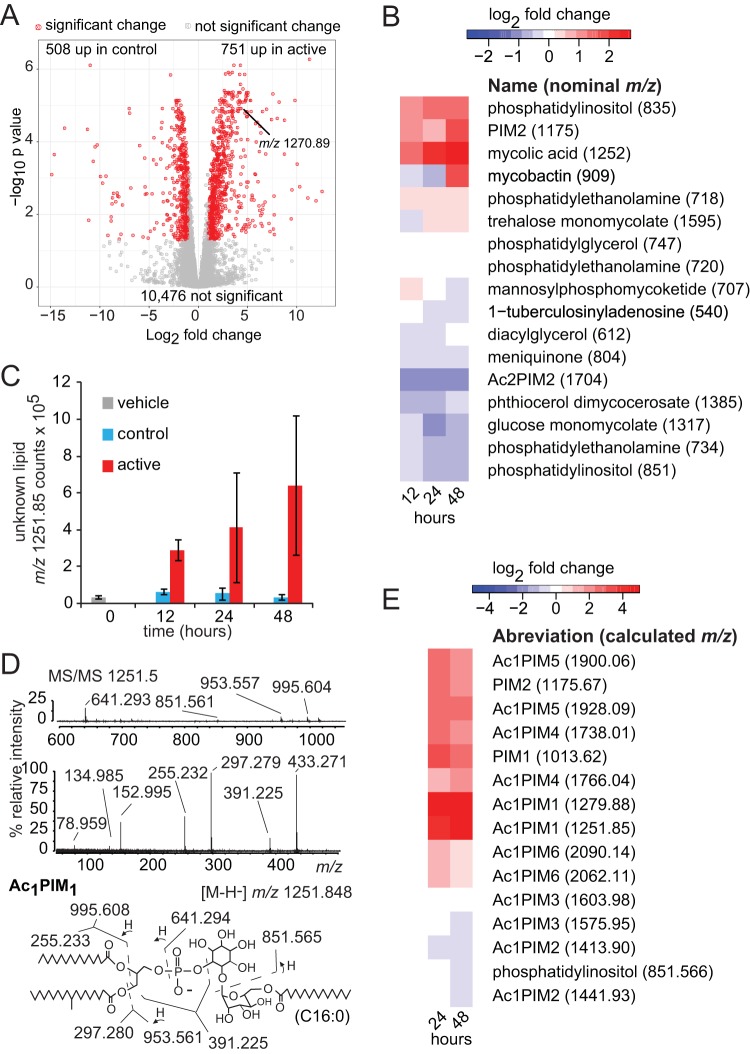
Lipidomics analysis. (A) Lipid extracts from *M. tuberculosis* treated with the kinase inhibitor or inactive control for 48 h were subjected to positive-mode mass spectrometry. Comparative lipidomics analysis detected 11,735 peaks corresponding to unnamed compounds of known mass (indicated in daltons). Significantly differentially abundant lipids (Log_2_FC, >1.0 or ≤1.0; *P* < 0.05) are indicated in red. Values represent results from three biological replicates and are representative of results from 3 independent experiments. (B) Retention time and mass values in MycoMap and MycoMass data sets were used to name ions corresponding to representative compounds among common classes of *M. tuberculosis* lipids. Analyzed using mean integrated peak areas, the patterns of change for all multiple molecular species in the same class were similar (not shown) over the 48-h treatment period. However, two molecular species of phosphatidylethanolamine with nominal molecular masses of 718 and 734 Da showed opposite responses. Greater than 2-fold enhancement of signals was seen for mycolic acid, phosphatidylinositol, and phosphatidylinositol mannosides (PIM) among the results of three independent experiments. (C) An unnamed ion with nominal *m*/*z* values of 1,270 in the positive mode (A) and 1,251 in the negative mode (C) showed a high fold change value, low *P* value (*P* < 0.005, *P* < 0.1, and *P* < 0.05 at 12 h, 24 h, and 48 h, respectively [two-tailed *t* test for comparison of inhibitor-treated samples to control treatment samples]) (A), and a time-dependent pattern of change after inhibitor treatment. (D) Collision-induced dissociation of the unknown established key components of acylated phosphatidylinositol monomannoside (Acyl1PIM). (E) Annotation and measurement of other members of the PIM family.

High-performance liquid chromatography-mass spectrometry (HPLC-MS)-based lipidomics identified over 10,000 unique *m*/*z* and retention time values (molecular features) in each experiment. Unbiased comparisons of unnamed molecular features (*n* = 11,735) showed that 1,259 (10.7%) met change criteria (Log_2_FC, >1.0; *P* < 0.05) after 48 h of treatment with the kinase inhibitor (Fig. 4A; see also Table S6). Features whose *m*/*z* and retention time data matched those of named lipids in MycoMap (43) allowed description of the lead molecule of 17 named lipid classes over time (Fig. 4B). Similarly to changes previously observed in response to hypoxic growth arrest (44), ions corresponding to the named lead molecules (Fig. 4B), as well as their chain lengths and unsaturation analogues, changed in parallel, indicating global regulation of all molecular variants in each lipid family. However, two molecular species of phosphatidylethanolamine (PE), which were deduced to contain C18:1 or C19:1 fatty acids, showed opposite responses to the inhibitor, demonstrating differential regulation results based on lipid length. Because these changes were time dependent, significant, and reproducible, these two forms of PE, named according to the nominal mass (PE 718 and PE 734), are listed separately (Fig. 4B).

10.1128/mBio.02333-17.9 TABLE S6  Abundance of named lipids at each time point following exposure to kinase inhibitor (A), abundance of PIMS at each time point (B), and abundance of TDM at each time point (C and D). Download TABLE S6, XLSX file, 0.03 MB. Copyright © 2018 Carette et al. 2018 Carette et al. This content is distributed under the terms of the Creative Commons Attribution 4.0 International license.

We noted a late but significant increase in mycobactin detection (Fig. 4B), consistent with the transcriptomic data. We also noted a marked, time-dependent increase of the abundance of free mycolic acids. Given the decreased expression of lipid synthases, this finding was unexpected and formed the basis of further validation experiments described in a separate section below.

Lipidomics also provides broad detection of lipids that are not annotated in MycoMap and other databases. Therefore, we interrogated all molecular features to identify unnamed molecules with large time-dependent change values. One feature with an *m*/*z* value of 1,270.89 and a retention time of 27 min (Fig. 4A) that showed marked, time-dependent increases (Fig. 4C) matched the expected mass of acyl phosphatidylinositolmannoside 1 (acyl PIM1). Collisional mass spectrometry yielded ions consistent with the expected fragments, including phosphatidylinositol (PI), acyl-hexose, and other diagnostic fragments (Fig. 4D). This finding prompted a more detailed interrogation of the PIMs, a family of glycolipids that differ in the number of acyl chains (X) and mannosyl residues (Y) that decorate the core PI structure (named "acyl X PIMY"). PIMs are important for cell membrane integrity, for maintaining the permeability barrier of the cell, and in cell division. They also have innate immune modulatory properties that are important for virulence (45). Detailed time course analysis revealed increases of many PIMs, especially those with three fatty acyl units and a small (one) or large (five) number of mannose units (Fig. 4E). Whether these changes in PIMs represent responses that are specific to kinase inhibition is not known as they have not been annotated in prior lipidome analyses. These changes in named lipids and in the newly identified acyl PIMs implicate PknA and PknB in regulating the lipid content of the mycobacterial cell envelope.

### Metabolomic links to gene expression for PG synthesis and lipid turnover.

**FIG 5:**
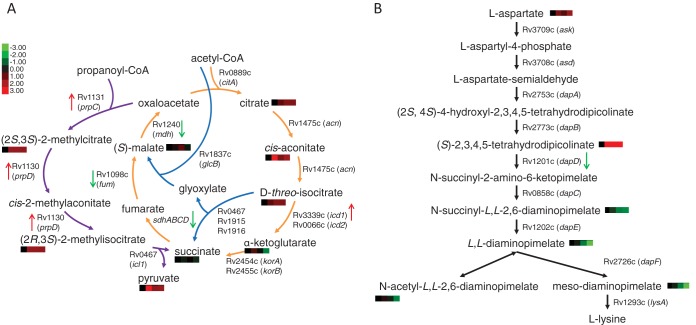
Impact of kinase inhibition on metabolic pathways. (A) Intermediates of TCA and methylcitrate cycles. (B) Intermediates of lysine biosynthesis. Relative metabolite abundances (based on ion intensities) in the presence of equimolar concentrations of either the kinase inhibitor or the inactive control are indicated in heat map format below or adjacent to each pathway intermediate detected in the metabolomics analysis; the 4 blocks indicate the durations of compound treatment (0, 12, 24, and 48 h), and color intensities correspond to Log_2_-fold difference relative to time 0 h; *P* = <0.05. Genes with a Log_2_FC value of >0.5 or ≤0.5 in the RNA-Seq results are represented with an arrow next to the gene, with red arrows pointing up to signify increased expression and green arrows pointing down to signify decreased expression. Primary data are shown in Table S2 (transcriptomics) and Table S7 (metabolomics).

Using an HPLC-MS analytic platform optimized for detection of polar small-molecule metabolites (46, 47), we focused on time-dependent changes in annotated intermediates of central carbon metabolism and PG precursor synthesis (Table S7). We noted a marked enrichment of intermediates associated with the first half of the tricarboxylic acid (TCA) cycle and with glyoxylate metabolism and the methylcitrate cycle and also noted step-specific changes in intermediates of lysine biosynthesis (Fig. 5).

10.1128/mBio.02333-17.10 TABLE S7  Metabolite abundance at each time point following exposure to kinase inhibitor (A), metabolite pathway enrichment analysis results (B), TCA and methycitrate cycle metabolite abundance in antibiotic-treated samples (C), and lysine biosynthesis metabolite abundance in antibiotic-treated samples (D). Download TABLE S7, XLSX file, 0.04 MB. Copyright © 2018 Carette et al. 2018 Carette et al. This content is distributed under the terms of the Creative Commons Attribution 4.0 International license.

Based on the known roles of acetate and propionate in the metabolism of unbranched and branched-chain fatty and mycolic acids, the observed increases in metabolites in the TCA and methylcitrate pathways would be expected after increased catabolism of *M. tuberculosis* cell wall lipids (Fig. 4B). Leveraging our multisystem approach, we identified matching changes in gene expression from our RNA-Seq data. These data specifically revealed markedly increased expression of the *prpD* (Rv1130) and *prpC* (Rv1131) methylcitrate cycle genes, suggesting that the increased levels of methyl(iso)citrate were indicative of an increased level of flux and, hence, of catabolism of odd chain fatty acids through the methylcitrate cycle (Fig. 5A; see also Table S2). These changes are distinct from the effects on central carbon metabolism observed in response to treatment with antibiotics (Table S7C) (48), but the accumulation of methylcitrate cycle metabolites in response to fatty acid or cholesterol catabolism has also been observed (49, 50).

Similarly, changes in levels of intermediates of lysine biosynthesis, which furnishes the PG precursor diaminopimelate (DAP), were interpreted to be indicative of structurally linked changes in the PG layer. We noted accumulation of (S)-2,3,4,5-tetrahydropicolinate and reduction in levels of downstream lysine and DAP intermediates. This finding led us to examine the RNA-Seq data, where we identified a significant decrease in expression of *dapD* (Rv1201c), which encodes tetrahydrodipicolinate N-succinyltransferase (Fig. 5B; see also Table S2). These findings have not been seen in metabolic profiles of *M. tuberculosis* treated with isoniazid, rifampin, or streptomycin and so argue against their being representative of a general response to growth arrest (Table S7D) (48). The linked metabolomics and transcriptomic results thus putatively identify *prpDC* and *dapD* as downstream components of the PknA and PknB network that more broadly appears to regulate mycobacterial cell envelope lipid and PG turnover.

### Multisystem analysis links Thr phosphorylation to an essential two-component system.

Our phosphoproteomics data identified a phosphorylation site at Thr^213^ in the DNA binding domain of the MtrA response regulator whose abundance was significantly decreased in the inhibitor-treated samples (Log_2_FC, −0.72; *P* = 5.1 × 10^−18^) (Fig. 2A; see also Table S3D), suggesting a previously unknown role for Thr phosphorylation in regulating MtrA activity. We therefore examined expression of MtrA-regulated genes, including the Rv1886c-Rv1884c (*fbpB*-Rv1885-*rpfC*) operon (51), which encodes the mycolyl transferase FbpB, a chorismate mutase homologue, and the PG lytic transglycosylase RpfC. These genes showed early and markedly decreased expression, with *fbpB* having the greatest decrease in expression of any gene at 48 h (Table S2). The genes with the next largest decreases in expression, Rv0950c and Rv2190c, encode predicted PG endopeptidases and are regulated by MtrA in the related actinomycete *Corynebacterium glutamicum*, indicating that they are likely to be regulated by MtrA in *M. tuberculosis* (52). Genes for RpfB and RipA, two additional PG lytic transglycosylases regulated by *M. tuberculosis* MtrA (53, 54), also showed strongly decreased expression (Table S2). With the exception of Rv2190c, these known or probable MtrA-regulated genes were linked to MtrA binding sites by chromatin immunoprecipitation sequencing (ChIP-Seq) analysis (55). Thus, our multisystem analysis suggested a previously unknown mechanism by which Thr phosphorylation of MtrA could control mycolyl lipids and PG. Therefore, we undertook targeted experiments focusing on two key aspects of this hypothesis.

### Thr^213^ phosphorylation disrupts MtrA binding to the *fbpB* promoter.

**FIG 6:**
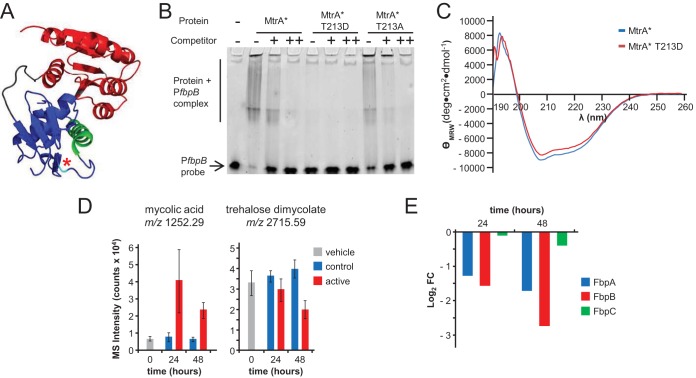
Analysis of native and Thr^213^-substituted MtrA*. (A) Previously determined structure of MtrA (56), rotated to show the position of Thr^213^ (cyan with asterisk) in a loop positioned at the carboxy terminal with respect to the DNA binding helix (green). (B) Data from EMSAs showing binding of MtrA* (MtrA Y102C) to the *fpbB* promoter, with decreased binding in the presence of specific competitor DNA (molar probe/competitor ratios of 1:50 and 1:1,000 for + and ++, respectively). The data also shown nearly complete elimination of binding of MtrA* containing the phosphomimetic substitution T213D but not of binding of MtrA* containing the nonphosphorylatable substitution T213A. Data are representative of results from at least 4 experiments. (C) Circular dichroism of MtrA* and MtrA* T213D. The same profile was obtained for both, showing that MtrA* T213D and MtrA* have highly similar folds. (D) Increased levels of free mycolates (*P* < 0.05 and *P* < 0.001 at 24 h and 48 h [two-tailed *t* test]) and decreased levels of trehalose dimycolates (*P* = 0.5 and *P* = <0.01 at 24 h and 48 h [two-tailed *t* test]) in kinase inhibitor-treated samples compared to control-treated samples. Data are from two independent experiments, each performed with biological triplicates (*n* = 6). (E) Time-dependent decreased abundance at 24 h and 48 h of the mycolyl transferase proteins FbpA (adjusted *P* = <1E−17 and <1E−24) and FbpB (adjusted *P* = <1E−16 and <1E−26) but not FbpC (adjusted *P* = 0.82 and <0.01) in response to the kinase inhibitor. Proteomic data (Table S5) were obtained from 3 independent experiments, each performed with biological triplicates.

Crystallography shows that Thr^213^ is in a loop immediately carboxy terminal to the DNA recognition helix of MtrA (Fig. 6A) (56), suggesting that Thr^213^ phosphorylation might affect DNA binding by MtrA. In both *M. tuberculosis* and *C. glutamicum*, MtrA acts as a repressor of the MtrA-regulated genes noted above. Our data thus suggest the hypothesis that MtrA Thr^213^ phosphorylation interferes with DNA binding and that decreased phosphorylation resulting from kinase inhibition leads to increased MtrA binding and repression of MtrA-regulated genes.

To investigate this hypothesis, we examined MtrA binding to a *fbpB* promoter region that contains a known MtrA binding site (51). We used an MtrA gain-of-function variant (MtrA containing a Y102C substitution [designated MtrA*]) that, unlike the native protein, does not require Asp^56^ phosphorylation of its receiver domain to bind DNA (57). We first verified specific binding of recombinant MtrA* to the *fbpB* promoter (Fig. 6B). We then utilized MtrA* proteins incorporating phosphomimetic (T213D) or nonphosphorylatable (T213A) substitutions to investigate the likely effect of phosphorylation of Thr^213^ on DNA binding. T213A-substituted MtrA* bound the *fbpB* promoter to an extent similar to that seen with MtrA*, whereas the T213D-substituted protein, confirmed by circular dichroism to be folded similarly to the native MtrA*, showed minimal binding at the same and higher concentrations of MtrA* that achieved complete probe binding (Fig. 6B and C and data not shown). These *in vitro* findings strongly support our hypothesis that decreased Thr phosphorylation of MtrA *in vivo* results in increased DNA binding, leading to increased repression of the *fbpB* operon and other regulated genes.

### Regulation of mycolic acid and mycolyl glycolipids.

The MtrA hypothesis intersected with several unexplained findings related to mycolyl lipids, which are an abundant and essential component of the mycobacterial outer membrane. We observed decreases in the levels of transcripts for key enzymes in the malonyl coenzyme A (CoA) and FASII pathway (encoded by *fasI*, *accA3*, *accD6*, *fabD*, *fabH*, *kasA*, and *kasB*) needed for *de novo* mycolic acid biosynthesis as well as increases in the levels of metabolites associated with mycolic acid catabolism (Table S2; see also Fig. 5). Yet we observed that mycolic acid was one of the lipids whose abundance was most highly increased (Fig. 4B). This counterintuitive result might be explained by mycolic acid release (recycling) from large glycosylated mycolate pools comprised of arabinomycolate, trehalose dimycolate (TDM; cord factor), and other mycolyl glycolipids, together with decreased incorporation of mycolates into the glycolipid pool. Free mycolic acid represents approximately 2% of the total cellular mycolate pool (T.-Y. Cheng and D. B. Moody, unpublished data). Therefore, even small increases in mycolate recycling or reductions in mycolate transfer to glycolipids could account for increased levels of free mycolic acids, as was previously observed during hypoxia-mediated bacteriostasis (44). Further, our data suggest that decreased levels of PknA/B-mediated MtrA Thr phosphorylation cause markedly decreased abundance of the mycolyl transferase FbpB, an effect that is predicted to increase the ratio of free to glycosylated mycolates.

*M. tuberculosis* has two additional genes encoding mycolyl transferases: *fbpA*, which also showed significantly decreased expression, and *fbpC*, which showed increased expression (Table S2). At the protein level, FbpA and FpbB levels were markedly decreased, but that of FbpC was only minimally changed (Fig. 6E). FbpC esterifies trehalose with mycolic acids to produce trehalose monomycolates (TMM) (58), whereas FbpB and FbpA show greater activity in converting TMM into TDM. Therefore, a unifying hypothesis is that unphosphorylated MtrA represses *fbpB* expression, which, together with decreased *fbpA* expression, leads to decreased transfer of free mycolates to TMM and finally to TDM. Because our standard lipidomics method is tuned to detect molecules with an *m*/*z* value of <2,000, we developed specialized electrospray methods to detect TDM (~2,500 *m*/*z*), which demonstrated a time-dependent, statistically significant decrease in TDM levels during kinase inhibitor-induced bacteriostasis (Fig. 4B and 6D). Thus, our multisystem data show decreased expression of pathways for *de novo* mycolic acid synthesis but a reduction in the levels of downstream mechanisms of mycolate transfer to glycosylated mycolate pools. Overall, these targeted experiments validated key aspects of a mechanism that emerged from our multisystem analysis: PknA/PknB-mediated Thr phosphorylation controls MtrA, which in turn regulates mycolate incorporation into glycosylated mycolates.

## DISCUSSION

To investigate the cellular functions of the essential *M. tuberculosis* transmembrane Ser/Thr protein kinases PknA and PknB, we undertook a multisystem analysis of responses to inhibition of these kinases. First, phosphoproteomic analysis identified highly significantly decreased phosphorylation at sites in 48 proteins, predominantly at Thr, the preferred target residue of both kinases (21). Newly identified phosphoproteins with decreased phosphorylation in response to inhibition of PknA and PknB are candidate novel substrates of these kinases that can be investigated in depth in future targeted analyses. Decreased phosphorylation of PG regulators, cell wall lipid metabolism and transport enzymes, cell division proteins, and several membrane proteins of unknown function suggests direct and extensive regulation of the cell envelope by PknA and PknB. Decreased phosphorylation of multiple protein secretion system proteins suggests a role for these kinases in controlling the *M. tuberculosis* secretome, a previously unknown regulatory target of these kinases.

**FIG 7:**
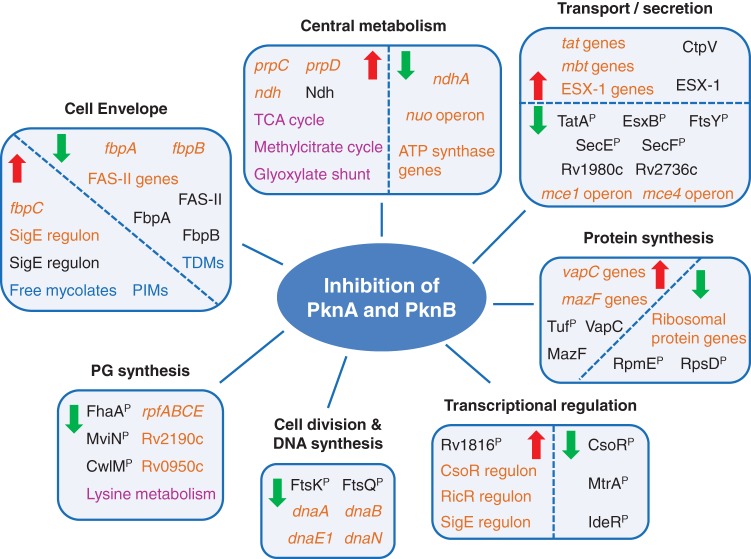
Major physiologies affected by inhibition of PknA and PknB. Selected highly significant molecular changes in response to inhibition of PknA and PknB are indicated. Changes in gene expression (orange), protein abundance (black), protein phosphorylation (black with superscript P), lipids (blue), and metabolites (purple) are shown. The red, upward-pointing arrows indicate molecules or pathways that increased in abundance and the green, downward-pointing arrows indicate molecules or pathways that decreased in abundance in inhibitor-treated samples compared to control-treated samples.

Beyond changes in protein phosphorylation, the multisystem effects of PknA and PknB inhibition represent indirect, downstream changes in response to kinase inhibition. Some of these changes appear to be specific to kinase inhibition, whereas others are seen in response to other causes of growth arrest. As we have highlighted, these multisystem effects indicate that these kinases impact an extensive range of physiological processes that determine the molecular structure of the mycobacterial cell envelope and that are required for the transmembrane transport of proteins, lipids, and other molecules (Fig. 7; see also Tables S2 to S7 in the supplemental material). These findings thus implicate PknA and PknB in regulating the bacterial interface with the extracellular environment.

The markedly increased expression of *sigE* and its regulon provides another indicator of perturbation of cell-environment interfaces in response to kinase inhibition. SigE is required for virulence and responds to cell envelope stresses through regulation of effector genes, including chaperones and proteases, and as part of a regulatory network that includes other sigma factors, transcription factors, and two-component systems (59–62). Similarly, the marked increase in copper-regulated gene expression suggests the presence of excess cellular levels of this metal, which may result from changes in cell envelope integrity.

Inhibition of PknA and PknB blocks *M. tuberculosis* growth, and our multisystem data provide evidence for coordinated changes in several pathways required for growth. These include decreased expression of ATP synthesis and electron transport pathways as well as decreased gene expression and protein abundance for most ribosomal proteins, changes that are seen in response to other conditions that lead to growth arrest. Decreased phosphorylation of ribosomal proteins S4 and L31, a novel finding of this work, also suggests a possible role for these kinases in regulating translation. We also observed significantly increased expression of several genes encoding VapC and MazF toxins in response to PknA/PknB inhibition (Table S2). These toxins block translation by cleaving RNAs required for protein synthesis in response to a variety of stresses (63, 64). This unexpected finding suggests a distinct mechanism of translation regulation linked to PknA and PknB kinase activity that is likely to be important during infection under conditions where *pknA* and *pknB* expression is repressed.

Regulation by PknA and PknB of *M. tuberculosis* growth and of the interface between the bacterium and the extracellular environment suggests a major role for these kinases in *M. tuberculosis* pathogenesis. Expression of *pknA* and *pknB* is maximal during logarithmic growth and decreases during the stationary phase, starvation, and hypoxia (11, 65, 66). The activity of these kinases is therefore likely to be critical during unrestricted growth early during infection and in regulating transitions between nonreplicating persistence and active growth. Conversely, decreased kinase activity and many of the resulting downstream responses to kinase inhibition described in this work are likely to occur under conditions where growth is restricted, such as at sites of caseous necrosis or in hypoxic granulomas during latency.

The intersection of the PknA/PknB pathway with the MtrA/B two-component system demonstrates unexpected connections among transcriptional, protein, and metabolic pathways that provide multipronged evidence for the existence of a novel regulatory mechanism. Our multisystem data support a model in which PknA/PknB inhibition causes decreased Thr phosphorylation of MtrA, resulting in increased DNA binding and marked repression of the MtrA regulon (Fig. 6). These effects would include markedly decreased expression of MtrA-regulated genes encoding PG degradation enzymes and the mycolyl transferase gene *fbpB*, which leads to accumulation of mycolic acids and decreases in levels of TDM products.

Extensive effects on the content of the mycobacterial cell envelope are highlighted by changes in levels of mycolates, phospholipids, and the newly named acyl PIMS identified in the lipidome analysis. Several other changes that likely affect mycolate dynamics are also represented in our data. These include decreased phosphorylation of the mycolate flippase MmpL3 (67), decreased expression of genes encoding FASII enzymes, increased expression of pathway intermediates involved in the degradation of fatty and mycolic acids, and decreased expression of the Mce-1 lipid transport system, all of which may contribute to changes in the levels of free and conjugated mycolates in response to PknA and PknB activity.

In addition to the pathways that we have highlighted in the text, the linked multisystem data sets produced in this work demonstrate many additional effects of inhibiting PknA and PknB. These data provide a valuable resource for investigators to develop new hypotheses regarding PknA and PknB function and represent ways in which regulatory and metabolic pathways intersect in the *M. tuberculosis* cell. In the context of the ongoing global tuberculosis epidemic and increasing rates of drug-resistant tuberculosis, new drugs to treat drug-resistant tuberculosis and to shorten treatment in cases of drug-susceptible tuberculosis are urgently needed. Given the clinical efficacy of several drugs that inhibit human protein kinases, our multisystem analysis of PknA and PknB inhibition highlights these essential kinases as promising targets for tuberculosis drug development.

## MATERIALS AND METHODS

### Primers used in this study.

Primers used in this study are listed in Table 1.

**TABLE 1:** Primer sequences used in the study

Name	Sequence
XC202	5′-ACACCATGGACCACCACCACCACCACCACACCATGAGGCAAAGGATTTT-3′
XC203	5′-AAGCTTTCACGGAGGTCCGGCCTTGT-3′
XC208	5′-GTCGGGCGCCGACGACTGCATCATGAAGCCGTTC-3′
XC209	5′-GAACGGCTTCATGATGCAGTCGTCGGCGCCCGAC-3′
XC210	5′-GGATCCCGAGAACCCGGCTGTGGTGCTGACCGTTCG-3′
XC211	5′-CGAACGGTCAGCACCACAGCCGGGTTCTCGGGATCC-3′
XC212	5′-GGATCCCGAGAACCCGGATGTGGTGCTGACCGTTCG-3′
XC213	5′-CGAACGGTCAGCACCACATCCGGGTTCTCGGGATCC-3′
XC220	5′-GGAGGCCAAATGTCGATTCGGGCGCAAAGTCGTCTCATTTCCGTATCGGTTACCGCC-3′
XC221	5′-GGCGGTAACCGATACGGAAATGAGACGACTTTGCGCCCGAATCGACATTTGGCCTCC-3′

### *M. tuberculosis* filter-based culture.

*M. tuberculosis* H37Rv or derivatives of this strain were used in all experiments. *pknD* and *pknL* deletion strains were constructed using a double-counterselection method as previously described (68), with confirmation of the mutation by DNA sequencing. *M. tuberculosis* was grown at 37°C using Middlebrook 7H9 with 5 g/liter bovine serum albumin, 2 g/liter glucose, and 0.85 g/liter NaCl (7H9-ADN) and 0.05% Tween 80 to an optical density at 600 nm (OD_600_) of 0.5. Using this culture, two cultures (one in 7H9-ADN plus 0.05% Tween 80 and the other in 7H9-ADN) were inoculated at an OD_600_ of 0.05 after two washes in 7H9-ADN. When the Tween 80-containing culture attained an OD_600_ of 0.3, *M. tuberculosis*-laden filters were prepared by passing 1 ml of the Tween-free culture by vacuum filtration through polyvinylidene difluoride (PVDF) membranes (Millipore catalog no. GVWP02500) (69). The filters were placed onto 7H9-ADN agar plus 1 g/liter casein (7H9-ADNC agar) plus 0.4% dimethyl sulfoxide (DMSO) and incubated at 37°C for 3 days. The filters were then transferred onto fresh 7H9-ADNC agar plus 0.4% DMSO for an additional 24 h at 37°C and then onto 7H9-ADNC agar containing the active inhibitor or the control compound at 20 μM unless otherwise specified. The filters were incubated at 37°C and harvested at 12, 24, or 48 h.

### Determination of inhibitor constant (*K*_*i*_) and MIC of inhibitor and control compounds.

The *K*_*i*_ of the active inhibitor and inactive analogue was determined for several *M. tuberculosis* STPKs (see Table S1 in the supplemental material). For PknA, PknB, and PknG, *K*_*i*_ values were determined using the specific substrate GarA (Rv1827). For PknD, PknE, PknF, PknH, and PknK, myelin basic protein was used as the substrate. *K*_*i*_ values were determined by fitting data to the Morrison tight-binding equation (70). Data were fitted using the Solver and XLfit modules of Microsoft Excel.

MICs of the kinase inhibitor and control compound were determined using the microplate alamarBlue assay (MABA) in 96-well microtiter plates, performed in triplicate as previously described (71). For MIC determination following *pknB* depletion, we used CRISPR interference vectors and procedures as previously described (20).

### Total protein preparation and phosphoproteomic and proteomic analysis. (i) Sample preparation, phosphopeptide enrichment (phosphoproteomics), and mass spectrometry.

Triplicate filter-based cultures were harvested at each time point. The bacteria were scraped from each filter directly into TRI reagent (Molecular Resource Center), and total protein extracts were prepared as previously described (21). Protein samples (300 µg) were prepared as previously described (72) with minor modifications and without isobaric labeling. Phosphopeptide enrichment was performed using a ProPac IMAC column (Fisher Scientific) as previously described (73) with minor modifications.

Samples were dissolved in 12 µl loading buffer (5% ACN, 5% formic acid) and sonicated for 5 min. Samples were placed in an autosampler linked to a nanoflow HPLC system (nanoLC 400; Eksigent) and a Q Exactive mass spectrometer. For peptide separation, a nano-cHiPLC Trap column (ReproSil-Pur C_18_-AQ; 200-µm inner diameter by 0.5-mm length, 3-µm pore size, 120 Å) and a nano-cHiPLC column (ReproSil-Pur C_18_-AQ; 75-µm inner diameter by 15-cm length, 3-µm pore size, 120 Å) were used. Peptides were eluted with a 60-min linear gradient from 93% buffer A (water with 0.2% formic acid) and 7% buffer B (acetonitrile with 0.2% formic acid) to 68% buffer A. The injection volume was 4 µl.

A Q Exactive mass spectrometer was run in positive-ion mode. Full scans were carried out at a resolution of 70 K with an automatic gain control (AGC) target of 3 × 10^6^ ions and a maximum injection time of 120 ms, using a scan range of 350 to 2,000 *m*/*z*. For tandem MS (MS/MS) data acquisition, a normalized collision energy value of 27 was used. Scans were carried out at a resolution of 35 K with an AGC target of 3 × 10^6^ ions and a maximum injection time of 120 ms. The isolation window was set to 2 *m*/*z*. An underfill ratio of 0.5% was set and a dynamic exclusion value of 20 s applied.

### (ii) Data analysis—phosphoproteomics and proteomics.

Data analysis was performed using Maxquant (version 1.5.2.8) and H37RV version 2 protein sequence, downloaded from PATRIC (74). Phosphorylation (STY) and oxidation (M) were used as variable modifications and carbamidomethylation as a fixed modification. Finally, the evidence file was used to extract the phosphopeptides. Using the modified sequence column, the average intensity of each modified peptide was calculated.

The phosphopeptides were normalized by the sum intensity value for each sample. To determine the effects of the active inhibitor versus the control compound on phosphopeptide abundance, we adapted metagenomeSeq (75), which accounts for the sparse nature of the detected phosphopeptides. Phosphopeptides that were detected in at least 7 of the 36 samples were included in the metagenomeSeq estimates for Log_2_FC, accounting for experimental batch effect as a covariate in the linear model. *P* values were adjusted using the Benjamini-Hochberg method.

Proteomic values were collected using a data-independent acquisition approach and normalized by the sum of the intensity values determined for each sample. Log_2_FC values and *P* values and Benjamini-Hochberg-adjusted *P* values for the comparison between the active inhibitor and control compound data at each time point were estimated using metagenomeSeq as described for the phosphoproteomics analysis. Protein expression enrichment for functional categories was performed on the Log_2_-transformed, quantile-normalized data using the Romer procedure as implemented in limma with 9,999 rotations, correcting for batch effects between experiments (76, 77).

To identify motifs in the set of unique phosphopeptides, we used *motif-x* (78), with the following parameters: central character, T, S, or Y; width, 13; number of occurrences, 20; background, all protein FASTA sequences from H37Rv version 2; significance; 0.0000417 (corresponding to a Bonferroni adjusted *P* value of 0.01); background central character, none. To identify motifs overrepresented in phosphopeptides that had significantly increased or decreased levels of phosphorylation after treatment with the inhibitor (Log_2_FC, >0.5 or ≤0.5, respectively; adjusted *P* = <0.001), we reran *motif-x* on the subset of phosphopeptides meeting these criteria. All *motif-x* parameters were the same as those used for the analysis of all phosphopeptides, except for the “number of occurrences” value, which was changed from 20 to 5 due to the smaller number of phosphopeptides that were significantly increased or decreased in abundance in response to the inhibitor.

### RNA extraction, RNA-Seq, and transcriptomic analysis.

Triplicate samples prepared for protein extraction as described above were used for RNA extraction. Total RNAs were extracted using a Direct-zol RNA MiniPrep kit (Zymo Research catalog no. R2052) according to the manufacturer’s instructions. The quantity and the quality of total RNAs were determined by measuring absorbance at 260 and 280 nm using a NanoDrop instrument (Thermo Fisher Scientific). The integrity of the total RNAs was assessed by the use of a Bioanalyzer in the BCH IDDRC Molecular Genetic Core. Two micrograms of total RNAs was subjected to rRNA depletion using a Ribo-Zero rRNA removal kit (Illumina catalog no. MRZB12424). The reaction cleanup was performed using an RNeasy minikit (Qiagen catalog no. 74104). The cDNA library was prepared using a NEBNext Ultra Directional RNA Library Prep kit for Illumina (New England Biolabs catalog no. E7420S), NEBNext multiplex oligonucleotides for Illumina (index primers set 1) (New England Biolabs catalog no. E7335S), and NEBNext multiplex oligonucleotides for Illumina (index primers set 2) (New England Biolabs catalog no. E7500S) according to the manufacturer’s instructions. The quantity and the quality of cDNA library were determined by Bioanalyzer analysis.

### Transcriptomic analysis.

RNA-Seq was performed at the Dana Farber Cancer Institute Center for Cancer Computational Biology (CCCB) core facility on an Illumina NextSeq 500 sequencer set to obtain 75-base single reads (SR75). RNA-Seq data were aligned using Spliced Transcripts Alignment to a Reference (STAR) software (79) to the H37RV version 2 genome sequence, downloaded from PATRIC (74). Reads that spanned noncanonical junctions, which may have a higher probability of being false positives, were removed. The featureCounts Subread package (80) was used to count reads. We applied normalization with trimmed means of M-values to the read counts using the edgeR R package (81). We applied the voom transformation to the read counts and performed linear modeling using the limma R package (77) to determine Log_2_FC values, *P* values, and Benjamini-Hochberg-adjusted *P* values comparing active inhibitor data to control data, treating batch effects as a covariate.

Gene symbols and gene products associated with Rv numbers were downloaded from the MTB Network Portal (http://networks.systemsbiology.net/mtb/). Gene ontology (GO) and KEGG Pathway functional categories were downloaded from PATRIC (74). We removed duplications and functional categories matching the regular expression \verb+eukary|plant|(?i)photosynth|E. Coli | bile | insect+. Gene expression enrichment for functional categories was performed on the Log_2_-CPM RNA-Seq data using the Romer procedure as implemented in limma with 9,999 rotations, correcting for batch effects between experiments (76, 77).

### Lipid extraction and lipidomic analysis.

Lipidomics analysis was accomplished as previously described (43) with minor modifications. Triplicate samples were harvested at serial time points by scraping the bacteria into 25 ml 1:2 (vol/vol) chloroform/methanol, followed by 1:1 (vol/vol) and 2:1 (vol/vol) chloroform/methanol to sterilize samples and extract lipids. The combined extracts were dried, weighed, and redissolved in the starting mobile phase (7:3 hexane/isopropanol) to make a total lipid concentration of 1.0 mg/ml. HPLC-MS was performed using a binary gradient (Agilent 1260) at a flow rate of 0.150 ml/min with detection using a time of flight mass spectrometer (Agilent 6530 Quadrupole Time-of-Flight [QTOF]) and electrospray ionization. Both positive-mode ionization and negative-mode ionization were used in separate analytic runs. Data were analyzed using Mass Hunter (Agilent), the R package XCMS for lipidomic peak analyses (82), and the R package limma for contrast-based analysis of significance (77).

### (i) Targeted analysis of polar phospholipids.

For targeted analysis of very polar lipids, including phosphatidylinositol mannosides, a reversed-phase method was developed using an Agilent Poroshell 120 (3-mm inner diameter by 50-mm length; C_18_) ultra-high-performance column with electrospray TOF MS detection in the negative mode, using 1-propanol and cyclohexane as the eluting mobile phase in solvent B, water and methanol in initial solvent A, and 2 mM ammonium acetate in both solvents. For higher levels of phosphatidylmannosides, up to the hexamannoside, a gradient of solvent A (1:19 water/methanol) and solvent B (9:1 1-propanol/cyclohexane) was run as follows: the initial eluent was 100% solvent B, and, after 4 min, the ratio was linearly increased to 100% solvent B at 14 min and held at 100% solvent B until min 18. To equilibrate for the next run, solvent A was run at 100% for 20 min.

### (ii) Targeted analysis of trehalose dimycolate.

For trehalose dimycolate analysis, lipids were resolved on the Poroshell 120 column using solvent A (1:9 water/methanol) and solvent B (7:3 1-propanol/cyclohexane) as described above. Chloroform (0.25 ml) was added to the lipid extract to improve lipid solubility followed by addition of solvent C to make a final concentration of 1 mg/ml. Runs started with 80% solvent B until min 2 with a linear increase to 100% solvent B at min 6, continuing at 100% solvent B until min 20, with a linear decrease to 80% solvent B at min 20 to min 22.

### Metabolite extraction and metabolomics analysis.

Triplicate samples were harvested at serial time points as described above. Metabolite extraction and metabolomics analysis were performed as previously described (48).

### Construction of *mtrA* encoding Y102C, Y102C-plus-T213A, and Y102C-plus-T213D substitutions.

Wild-type *mtrA* was obtained by PCR using *M. tuberculosis* H37Rv chromosomal DNA as the template and primers XC202 and XC203. The amplicon contained NcoI and HindIII restriction sites for cloning into pET28a to express an N-terminally His_6_-tagged MtrA. Mutations Y102C, T213A, and T213D were introduced by the use of a QuikChange XL site-directed mutagenesis kit (Agilent Technologies catalog no. 200516) and, respectively, primers XC208 and XC209, primers XC210 and XC211, and primers XC212 and XC213. All alleles were confirmed by sequencing of the entire gene.

### Production of recombinant MtrA and electrophoretic mobility shift assay (EMSA).

His_6_-MtrA proteins were produced in *E. coli* BL21 (DE3) pLysS (Novagen catalog no. 69451) as previously described (51). Protein folding of MtrA* and MtrA*T213D was analyzed by circular dichroism. The proteins were dialyzed against 20 mM phosphate–50 mM sodium (pH 8.0), concentrated to 10 μM, and placed in a stoppered 0.1-cm-path-length quartz cuvette. Three Far-UV spectra were generated for each protein as previously described (83). The values are expressed as mean residue molar ellipticity (ϴ_MRW_) in degrees per square centimeter per decimole.

Binding of the protein to the *fbpB* promoter region (P*fbpB*) was analyzed by EMSA using a Cy3-labeled P*fbpB* probe sequence (51). The probe and unlabeled competitor P*fbpB* were prepared by annealing primer XC220 labeled with Cy3 at the 5′ end to XC221 labeled with Cy3 at the 5′ end) and XC220 to XC221, respectively. The reaction buffer consisted of 20 mM Tris-HCl (pH 7.5), 150 mM NaCl, 50 mM KCl, 10 mM dithiothreitol (DTT), 5 µM EDTA, 5% glycerol, 0.05% NP-40, 2 µg poly(dI) ⋅ poly(dC), 200 fmol probe, and 100 pmol MtrA (probe/protein molar ratio of 1:500). The samples were incubated for 30 min at room temperature and resolved by 7.5% TG native gel electrophoresis (120 V, 4°C, 60 min). The gel was scanned using a Kodak Image Station 4000MM system.

### Data and material availability.

Transcriptomics data are in Table S2 and are deposited into the Gene Expression Omnibus (GEO) database under the accession number GSE110508. Proteomics data are in Tables S3 and S5 and are deposited to the ProteomeXchange Consortium via the PRIDE partner repository with the dataset identifier PXD008968. Lipidomics data are provided in Table S6. Metabolomics data are provided in Table S7.
