# Supplementary material for: Multisystem Analysis of Mycobacterium tuberculosis Reveals Kinase-Dependent Remodeling of the Pathogen-Environment Interface
Source: mBio. 2018 Mar 6;9(2):e02333-17. doi: 10.1128/mBio.02333-17 (PMC5845002; doi:10.1128/mBio.02333-17)
Supplement: TABLE S1 [file mbo001183756st1.docx]

| **Compound** | **Molecular**  **weight** | **Solubility**  **pH 7.4 (μM)** | **Ki (μM)** | | | | | | | | |
| --- | --- | --- | --- | --- | --- | --- | --- | --- | --- | --- | --- |
|  |  |  | **PknA** | **PknB** | **PknD** | **PknE** | **PknF** | **PknG** | **PknH** | **PknK** | **PknL** |
| **Inhibitor** | 375 | 8.6 | 0.018 | 0.004 | 0.003 | 0.036 | 0.46 | 0.165 | 0.024 | >5.0 | 0.006 |
| **Control** | 390 | 6.9 | >5.0 | >5.0 | >5.0 | >5.0 | >5.0 | >5.0 | >5.0 | >5.0 | >5.0 |

Table S1. Activity of kinase inhibitor and inactive control compounds against *M. tuberculosis* kinases.
