# Supplementary figures and images for: Multisystem Analysis of Mycobacterium tuberculosis Reveals Kinase-Dependent Remodeling of the Pathogen-Environment Interface
Source: mBio. 2018 Mar 6;9(2):e02333-17. doi: 10.1128/mBio.02333-17 (PMC5845002; doi:10.1128/mBio.02333-17)

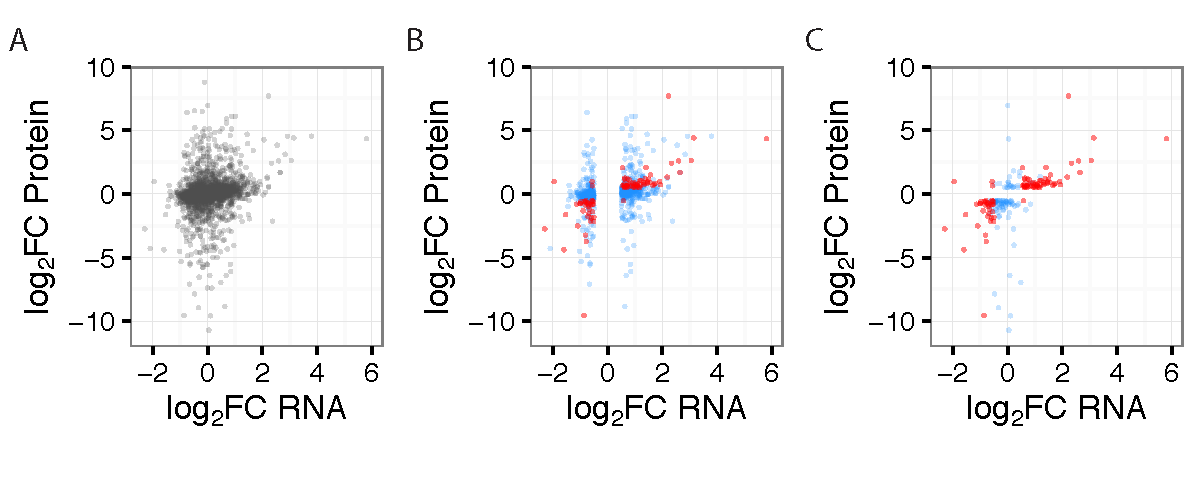

Supplement: FIG S3 [file mbo001183756sf3.tif]
