# Supplementary material for: Multisystem Analysis of Mycobacterium tuberculosis Reveals Kinase-Dependent Remodeling of the Pathogen-Environment Interface
Source: mBio. 2018 Mar 6;9(2):e02333-17. doi: 10.1128/mBio.02333-17 (PMC5845002; doi:10.1128/mBio.02333-17)
Supplement: Supplementary file 7 [file mbo001183756sf1.docx]

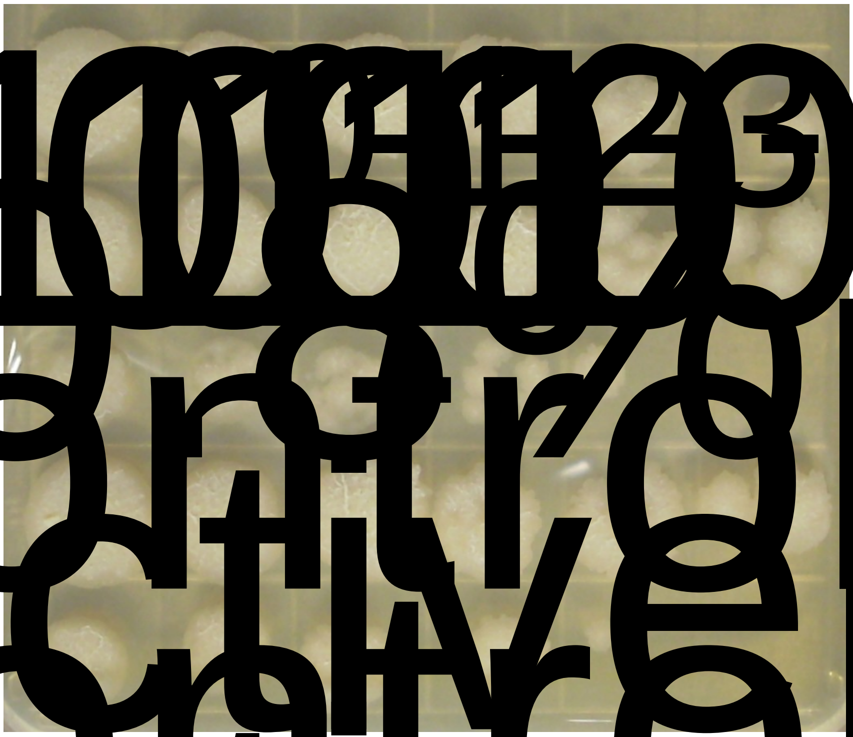

[illegible]

control [illegible]

active [illegible]

control [illegible]

active [illegible]
